# How to use your eye medication

**Published:** 2023-05-22

**Authors:** Mwangi Nyawira, Leah Kenan

**Affiliations:** Deputy Director: Academics, Kenya Medical Training College, Nairobi, Kenya.; Patient Care Manager: City Eye Hospital, Nairobi, Kenya.

**Once you have your medication, what is the procedure you should follow?**

**Figure:**
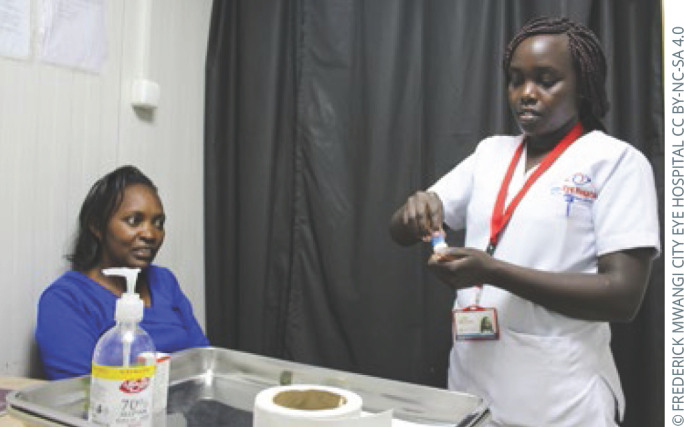
Opening the eye drops. kenya

## Before you start

1. Assemble the items you need: the medication, clean tissue paper or paper towel, mirror, soap and water, or hand sanitiser (note: hand washing is preferred).
2. Read the written instructions on how to use each of the medications. This could be on the bottle, on the package insert, or on a separate sheet given to you at the hospital or pharmacy.
3. If using a drop, gel, and/or ointment, use them in this sequence; drop first, then gel, and then the ointment.
4. If the medicine is for treating a problem on your eyelid, it should be administered on the eyelid and not in the eye.
5. Do not instil medication into an eye that has been injured. Visit the eye clinic for review of the injury as soon as possible.
6. If the eye has discharge, clean the eye using sterile cotton wipes dipped in cooled, boiled water or bottled water before instilling the medication.

## Preparation

1. Take off your spectacles if you are wearing any.
2. Wash your hands with soap and water and dry them using a paper towel. Hand washing is preferred, but if you are in a situation where this is not possible, you can use hand sanitiser instead.
3. If you are wearing contact lenses, take them out — unless your doctor has told you to leave them in.
4. Place a clean tissue or paper towel on the nearest surface.
5. If using eye drops, shake the bottle gently.
6. Remove the cap and place on the tissue or paper towel. Do not touch the tip. Make sure the dropper (or tube) and the cap stay clean.

If you are instilling the drops yourself:

1. Tilt your head back, open both eyes and look up towards the ceiling. Focus on a specific point on the ceiling. Do not blink or wipe the eye.
2. With the thumb and forefinger, pull your lower lid down and outwards to expose the lower conjunctival sac: the pocket where you instil the medication. You can stand in front of a mirror, or ask someone to hold a smaller mirror for you.
3. Hold the dropper bottle or tube between the thumb and index finger of your other hand. (If someone is helping you, they can hold the dropper bottle or tube and instil the drops or ointment.)
4. Position it so the tip is directly over the eyelid pocket.
5. Ensure the tip is not touching the eye, eyelids, or eyelashes; this avoids contaminating the drug.
6. Squeeze the bottle or tube gently to instil the eye drop (just one drop) or gel into the pocket (conjunctival sac), as instructed. Avoid letting the medication make contact with the sensitive cornea.

## Getting the dosage correct

1. You may feel the cold drop or gel/ointment once it enters the eye, especially if the medication was stored in the refrigerator.
2. As long as you feel the medication in your eye or on the edge of the eyelid, you've been successful. You don't need to add more than one eye drop. The drop may spill over onto your cheek; this is okay as long as you felt it hit the surface of your eye.
3. If the medicine falls on your face or clothes or floor, but not actually into the eye, try again.
4. After instilling the drop, shut the eyelids lightly for a few seconds (up to one minute) while pressing your finger lightly on the inner corner of our eye, nearest your nose. This is known as the canthus and is the location of your tear ducts (visible as small holes). Doing this prevents the medicine draining into your nose, which improves absorption of the medicine where it is needed most – in your eye. Try not to blink.
5. If any liquid drops leaked out from your closed eyelids, blot around your eyes using a clean tissue or paper towel to remove the excess.
6. Repeat the steps with the other eye, if instructed to do so.
7. If using more than one type of eye drop, wait for 5–10 minutes between each of the different medications.
8. Wash hands with soap and water when done.

**Figure:**
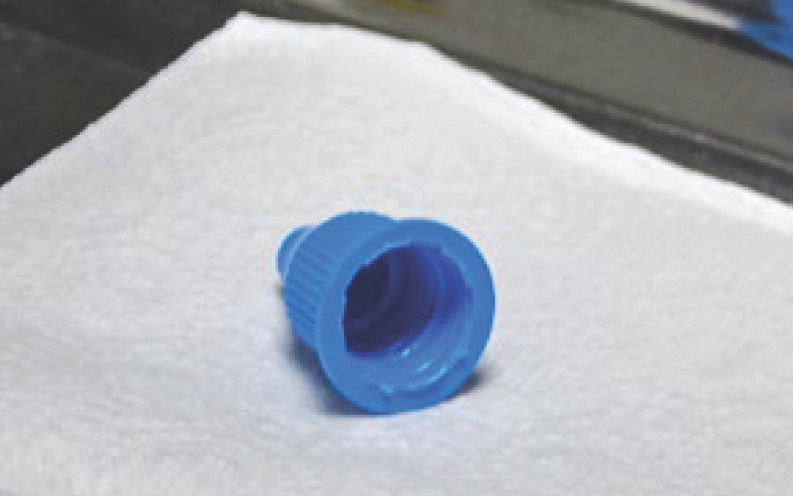
Correctly placing the cap on a clean paper towel.

**Figure:**
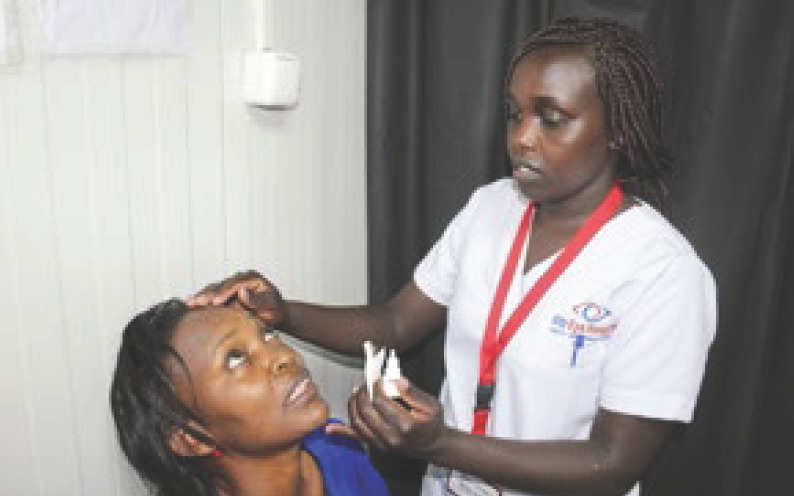
Positioning the patient.

**Figure:**
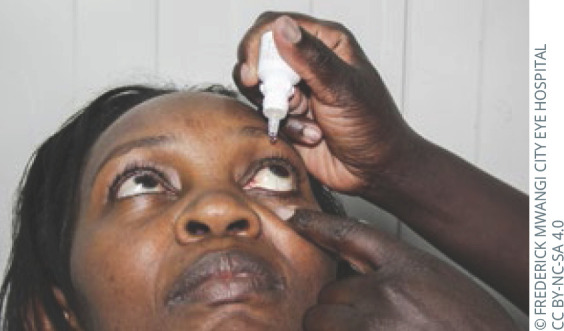
Instilling eye drop in the conjunctival sac.

